# An International Survey-based Algorithm for the Pharmacologic Treatment of Obsessive-Compulsive Behaviors in Huntington’s Disease

**DOI:** 10.1371/currents.RRN1261

**Published:** 2011-09-20

**Authors:** Karen Anderson, David Craufurd, Mary C. Edmondson, Nathan Goodman, Mark Groves, Erik van Duijn, Daniel P. van Kammen, LaVonne Goodman

**Affiliations:** ^*^Department of Psychiatry and Department of Neurology, University of Maryland, School of Medicine, Baltimore, MD USA; ^†^University of Manchester, Manchester Academic Health Sciences Centre and Central Manchester University Hospitals NHS Foundation Trust, Manchester, UK; ^‡^Department of Psychiatry, Duke University Medical Center & North Carolina Center for the Care of Huntington's Disease; ^§^Institute for Systems Biology, Seattle, WA; ^¶^Departments of Neurology and Psychiatry, Beth Israel Medical Center, New York, NY; ^#^Department of Psychiatry, Leiden University Medical Centre, Leiden; and Centre for Mental Health Care Delfland, Delft, Netherlands; ^**^Formerly CHDI Foundation, Inc. Presently independent CNS development consultant and ^††^Huntington's Disease Drug Works, Lake Forest Park, WA

## Abstract

It is generally believed that treatments are available to manage obsessive-compulsive behaviors (OCB's) in Huntington’s disease (HD). However, lack of an evidence base prevents guideline development. The research literature fails to address the indications for behavioral interventions, drug selection, drug dosing, management of inadequate response to a single drug, and preferred drugs when additional behavioral symptoms comorbid to OCBs are present. In an effort to inform clinical decision-making, we surveyed an international group of experts to address these points. Survey results showed that experts utilized behavioral therapy only for patients with mild cognitive impairment. There was expert agreement that a selective serotonin reuptake inhibitor (SSRI) was the first choice drug, although clomipramine (CMI) was cited as a monotherapy choice by the smaller number of experts familiar with its use. Perceived efficacy for control of OCBs was similar for both SSRIs and CMI. Though less favored choices overall, antipsychotics (APDs) and antiepileptic mood stabilizers (AEDs) were most often used as augmentation strategies. In addition to survey results, this report reviews available studies, and lastly presents an algorithm for the treatment of OCBs in HD based on practice-based preferences obtained from this survey.

## **Introduction**

Huntington’s disease (HD) is an inherited neurodegenerative illness characterized by a combination of motor, cognitive and behavioral abnormalities. A wide variety of behavioral disturbances have been described in HD patients, including perseveration and obsessive-compulsive symptoms. For the purposes of this discussion we use the single term, obsessive-compulsive behaviors (OCBs) to encompass this entire range of these symptoms. Research into OCBs in HD is limited; however reports suggest that OCBs occur in 20 to 50% of HD patients [1] [2] [3] [4] [5] [6] [7]. OCBs have also been reported in prediagnosed individuals who carry the HD gene expansion [8] [9] [10]. Similarly, case reports have described instances of OCBs [11] [12], and an instance of obsessive gambling in an HD pedigree [13]. 

**Box 1. Interview Questions for Assessment of OCBs.**

**Table:** 

Do you get stuck on certain ideas that seem to go through your head over and over?
Do you like to have certain things done on a very definite schedule?
Are you worried about dirt, infections, contamination, more than other people?
Is it upsetting to you when things change unexpectedly?
Do you like to collect things, especially items that other people might find worthless (e.g., empty cologne bottles, worn out clothing, old newspapers)?
Do you like things arranged a certain way, for example, all the clothes in your closet must be in order by color? If so, do you get very upset if someone else moves things out of place?
Are there certain actions that you do over and over?
Do people say you ask the same questions over and over?

OCBs are problematic because they are often difficult to recognize. However, it is vital to identify these symptoms, because mischaracterization of these patients as difficult, stubborn, or "character disordered” adds greatly to the strain experienced by patients and families. If these behaviors are untreated, they can lead to management problems, including aggression toward care partners, family members, and medical staff. Untreated OCBs can greatly complicate long-term care and nursing home placement. Box 1 gives examples of questions for use in the evaluation of OCBs.

**Box 2. DSM IV criteria for obsessions and compulsions.**

**Table:** 

**Obsessions **
Recurrent and persistent thoughts, impulses, or images that are experienced, at some time during the disturbance, as intrusive and inappropriate and that cause marked anxiety or distress
The thoughts, impulses, or images are not simply excessive worries about real-life problems
The person attempts to ignore or suppress such thoughts, impulses, or images, or to neutralize them with some other thought or action
The person recognizes that the obsessional thoughts, impulses, or images are a product of his or her own mind (not imposed from without as in thought insertion).
**Compulsions **
Repetitive behaviors (e.g., hand washing, ordering, checking) or mental acts (e.g., praying, counting, repeating words silently) that the person feels driven to perform in response to an obsession, or according to rules that must be applied rigidly.
The behaviors or mental acts are aimed at preventing or reducing distress or preventing some dreaded event or situation; however, these behaviors or mental acts either are not connected in a realistic way with what they are designed to neutralize or prevent or are clearly excessive.
*Source*: (DSM-IV, American Psychiatric Association, 1994)

Information obtained from care partners about OCBs is invaluable in any assessment since insight into these symptoms is often limited in HD patients. OCBs can be a tipping point in terms of care partner burnout and subsequent need to institutionalize HD patients. Offering a care partner the opportunity to discuss OCBs can be part of a more general discussion on caring for the person with Huntington’s disease. The OCBs seen in most patients with HD do not meet DSM criteria for Obsessive-Compulsive Disorder (OCD) as defined by DSM IV (Box 2), because patients often lack insight into the symptoms and are not troubled by them. The majority of OCBs in HD are better defined as perseverative behaviors. Perseveration is the uncontrolled repetition or continuation of a response (motor act, word, thought, activity, strategy, or emotion) in the absence of an ongoing occasion or rationale for the behavior [14]. Perseveration often results from a disruption of frontal-subcortical circuitry in conditions such as traumatic brain injury, schizophrenia, autism, Alzheimer’s disease, and frontal dementias such as HD. 

  Even though the OCBs seen in most patients with HD do not fully meet DSM criteria for OCD, it is practical to group perseverative symptoms and obsessive and compulsive symptoms together because the behavioral and pharmacologic interventions are similar. Features of frontal lobe dysfunction, such as impulsivity, may complicate OCBs, as patients may have difficulty suppressing the urge to act on ideas or emotions, resulting in irritability or aggression. OCBs interfere with learning and adaptive behavior (interactions with others, on-task behavior, task shifting, mental flexibility). Pharmacologic treatment of perseverative behavior may “unblock” performance potential and result in improved overall functioning in activities of daily living [14].

**Box 3. Abbreviations for drugs and drug classes**

**Table:** 

AED	mood stabilizing anti-epileptic drug
APD	antipsychotic
BZD	benzodiazepine
CMI	chlomipramine
SNRI	serotonin-norepinephrine reuptake inhibitor
SSRI	selective serotonin reuptake inhibitor
TCA	tricyclic

 Lacking an adequate evidence base to guide treatment of OCB's in HD, we surveyed current clinical practice among an international group of HD experts to ascertain practice-based preferences. Recognizing the limits of expert opinion, and with the expectation that future clinical research will provide evidence-based information, we present survey results to provide direction for the management of OCBs in HD. 

## **Methods**

The OCB survey was one of three symptom surveys developed by three core groups of nine psychiatrists and neurologists drawn from the European Huntington’s Disease Network (EHDN), the Huntington Study Group (HSG), and an HD family representative. Concurrent surveys were developed for irritability and chorea in HD. These specific three symptoms were chosen by core member concensus as those in greatest need of expert guidance relative to other symptoms of HD including depression, anxiety, sleep disorder, and psychotic behaviors, for which clinical practice follows the guidelines developed for these conditions in the general population. Data on coincident surveys for the treatment of irritability behaviors and chorea are presented in separate articles [15] [16].

 The OCB survey was developed by five psychiatrists from different geographic regions, who have had extensive experience treating OCB symptoms in HD. Questions were constructed electronically. Subsequently the survey was distributed by email link to a larger international group of EHDN and HSG physician leaders from HD specialty centers in 11 European countries, 10 U.S.A states, 4 Canadian provinces, and 3 Australian states. Experts were selected by the combined EHDN and HSG core groups as being knowledgeable in treating HD behavioral symptoms. Follow-up email or telephone reminders were used to encourage survey participation. Respondents received a small honorarium after completing the survey.

 The OCBs survey consisted of 51 multiple-choice questions with 680 alternative answers, with the option to add comments. Questions addressed respondents' demographics and patterns of behavioral and pharmacological treatment. By core group consensus, the survey focused on the use of 5 drug classes (SSRIs, APDs, AEDs, benzodiazepines (BZDs), and tricyclic antidepressants (TCAs)) and 2 specific drugs (clomipramine (CMI), and lithium) that have been used to treat OCBs in HD. In iterative fashion, each drug/drug class was addressed separately through additional questions covering the following factors: patterns of use (first choice, alternative monotherapy, adjunctive therapy, not an appropriate use, insufficient experience), perceived effectiveness (very effective, effective, somewhat effective, minimally effective), and preferred drugs within each class. Branching logic utilized in the electronic survey prevented the answering of questions if a respondent did not choose a specific treatment as first or alternative monotherapy, or indicated having no experience with a particular treatment. Questions also covered augmentation and switch strategies, timing of dose titration, and preferred drugs when other behavioral symptoms comorbid to OCB's were present.

 Following analysis of survey data, the core group presented results and a proposed OCBs treatment algorithm to broader groups of international experts attending the 2010 EHDN and HSG annual meetings for the purpose of obtaining further expert opinion and review.   

## **Results**

Of the 66 expert clinicians contacted, 49 (75%) responded. Not all respondents answered all individual questions. Of these 49 experts, 21 were from Europe, 26 from North America, and 3 from Australia. Most respondents were neurologists (33) or psychiatrists (14), and 2 were double boarded in neurology and psychiatry. Most respondents reported seeing at least 50 HD patients per year; there were only 8 who reported seeing fewer than this number. Fourteen reported seeing 50-100 patients annually; 9 reported seeing 100-150 and 18 more than 150 patients per year.

**Behavioral Interventions**: The first questions concerned non-pharmacological interventions for OCBs; specifically, respondents were asked treatment practice using cognitive behavioral therapy (CBT) in patients with either mild, moderate, or severe cognitive impairment. There was also a query about the perceived benefit of family education. Many respondents (23%) indicated having no experience with CBT for HD patients, but others (50%) reported that CBT was at least somewhat effective, but only in those with mild cognitive impairment. It was not endorsed for patients with moderate or severe cognitive impairment. However, 83% of respondents endorsed family education on OCBs.

**Practice patterns by drug/drug class**: The next set of treatment questions concerned drug selection and was phrased as follows: “Assuming there are no comorbid symptoms to influence your decision, what is your practice pattern with the use of [drug or drug class] for the treatment of OCBs in Huntington’s disease?”

 An SSRI was first choice of most respondents for treatment of OCBs when no comorbid symptoms influenced treatment decisions. CMI was the next most frequently endorsed first choice, followed by APDs and AEDs. Considering all monotherapy choices (first and alternative) SSRIs were most frequently endorsed (89%). CMI also rated highly and was endorsed as monotherapy by 63% of respondents who had experience with this drug. The BZDs, TCAs (excluding clomipramine) and lithium were not chosen as first choice agent by any respondent. APDs and AEDs were often utilized as augmenting and adjunctive therapies. BZDs were most often used as adjunctive therapy when anxiety was a comorbid factor.

**Figure:**
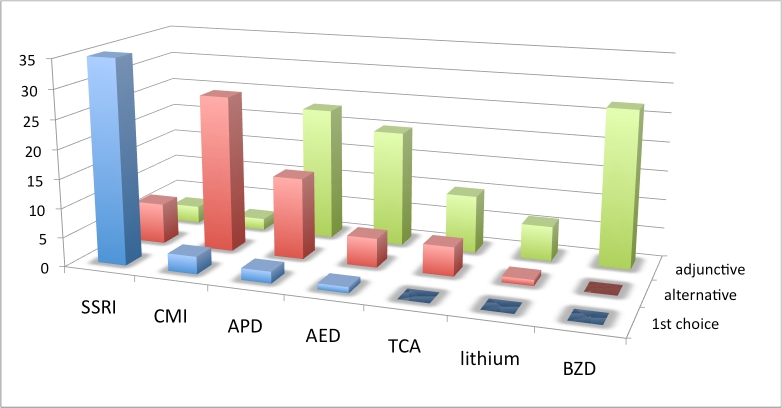
**Figure 1. Choice of drug from OCBs across all geographic regions. Vertical axis is number of responses. See box 3 for abbreviations.**

**Figure:**
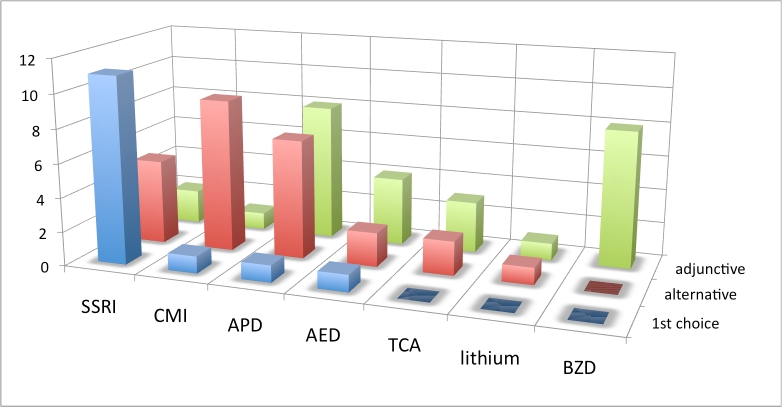
**Figure 2. Choice of drug for OCBs, European respondents. Vertical axis is number of responses.  See box 3 for abbreviations.**

**Figure:**
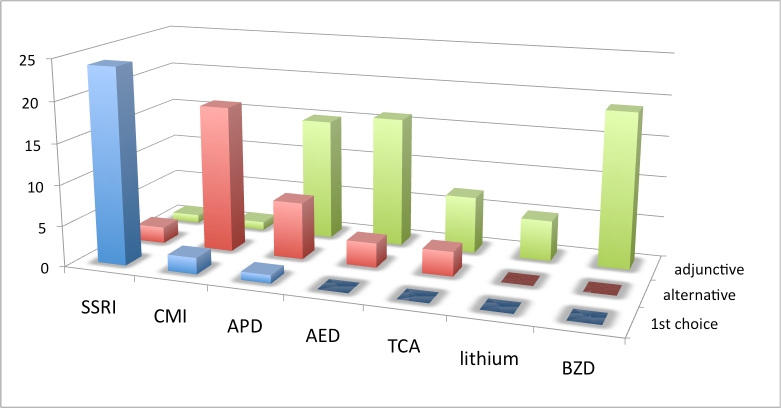
**Figure 3. Choice of drug for OCBs, North American and Australian respondents.  Vertical axis is number of responses.  See box 3 for abbreviations.**

**Table 1. Choice of drug for treating OCBs across all geographic regions. N is number of responses. Percentages are relative to N. See box 3 for abbreviations.**

**Table:** 

						
**SSRI**	47	74%	15%	6%	2%	2%
**CMI**	47	6%	57%	4%	4%	28%
**APD**	47	4%	30%	49%	15%	2%
**AED**	47	2%	11%	43%	17%	28%
**TCA**	47	0%	11%	21%	32%	36%
**lithium**	47	0%	2%	13%	28%	57%
**BZD**	47	0%	0%	57%	38%	4%

**Perceived efficacy of drug choice**: Most experts indicated that SSRIs are the most effective drug choice for treating OCBs in HD. For respondents experienced with clomipramine, efficacy ratings for this drug were similar to SSRIs. Table 1 summarizes expert views about the relative efficacy of the surveyed drugs. 

**Table 2. Expert opinion of drug efficacy for treating OCBs. N is number of responses. Percentages are relative to N. See box 3 for abbreviations.**

**Table:** 

					
**SSRI**	46	4%	39%	48%	9%
**CMI**	32	3%	41%	50%	6%
**APD**	40	0%	33%	60%	8%
**AED**	27	0%	7%	67%	30%
**TCA**	15	0%	20%	73%	7%
**lithium**	7	0%	0%	57%	50%
**BZD**	28	0%	4%	75%	21%

**Perceived benefit of high dose SSRI optimization**: Respondents were also asked about SSRI dosing optimization for treating OCBs in HD to upper limits of manufacturer recommended dosage for depression. Though all respondents perceived a level of increased effectiveness with higher dosing, the degree of perceived effect varied widely. Seven respondents reported beneficial results in selected patients using dosage exceeding that recommended by the manufacturer.  Subsequent to the survey, the Federal Drug Administration issued a directive to change manufacturer recommended high dosage of citalopram from 60 mg to 40 mg per day due to increase in heart arrhythmias and lack of benefit of the higher dose for treating depression.  However, seven respondents reported beneficial results for treating OCBs in selected patients using doses higher than that recommended for depression.

**Dosing interval choices**: The respondents were asked about dose titration intervals for the drug/drug class alternatives. For both SSRI and CMI, the top two pharmacological agents for monotherapy, more of respondents reported they would increase from initial dose after 4-6 weeks, followed by 2-4 weeks as the second most endorsed option (Table 3).

**Table 3. Choice of dosing titration intervals for drugs used to treat OCBs. N is number of responses. Percentages are relative to N. See box 3 for abbreviations.**

**Table:** 

					
**SSRI**	46	7%	28%	57%	8%
**CMI**	32	9%	38%	47%	6%
**AED**	26	15%	31%	50%	4%
**TCA**	15	0%	20%	60%	20%
**BZD**	27	41%	30%	19%	11%

**Adding or switching drugs for inadequate response to initial drug choice**: The next set of iterative questions regarded strategies for either adding or switching drug when an initial drug failed to adequately treat OCBs in HD. The most notable result to this set of questions is that no consistent pattern was demonstrated. When SSRI was chosen as initial monotherapy but gave no or only partial benefit, the next step in management varied: switch to another SSRI (23%), switch to CMI (18%), add CMI (16%) or an APD (16%), switch to serotonin and norepinephrine reuptake inhibitor (SNRI) (14%).  Less common choices (5% each) were: switch to an APD or add BZD. When CMI was chosen as initial monotherapy but gave no or only partial benefit, next step choices also varied and included: add an APD (41%), switch to SSRI (26%), add SSRI (11%), add an AED (11%), or switch to an APD (7%). Fewer than 5% chose any other option. In response to separate questions regarding APDs, AEDs, BZDs or TCAs (other than clomipramine), the majority of those surveyed reported they used all of these agents most frequently as adjunctive therapy. 

**Table 4. Alternate choice of drug for treating OCBs when inadequate response to initial therapy. N is number of responses. Percentages are relative to N. Alternate listed only if chosen by 5% or more of respondents. See box 3 for abbreviations.**

**Table:** 

			
**SSRI**	44	switch to another SSRI	23%
		switch to CMI	18%
		add CMI	16%
		add APD	16%
		switch to SNRI	14%
		switch to APD	5%
		add BZD	5%
**CMI**	27	add APD	41%
		switch to SSRI	26%
		add SSRI	11%
		add AED	11%
		switch to APD	7%

**Specific drugs favored within class**: Respondents were asked about preferred drugs within class for treating OCBs in HD. Preferred drugs included the SSRIs: citalopram (35%), sertraline (25%), paroxetine (15%), fluoxetine (11%), the APDs: olanzapine (52%), risperidone (34%), quetiapine (23%), aripiprazole (21%), the AEDs: valproate derivatives (70%), carbemazepine (22%), lamotrigene (16%), topiramate (11%). When using BZDs, favored drugs were: clonazepam (60%), alprazolam (28%), lorazepam (27%). For a separate query regarding Lithium, most respondents reported insufficient experience using this agent for OCBs in HD (56%). Another 29% felt it was not an appropriate alternative. Only 14% reported using it as monotherapy.

**Preferred drug for OCBs given comorbid psychiatric symptoms**: Respondents were asked, for each of the medication classes (SSRI, CMI, APBs, AEDs, BZDs, TCAs, lithium) about how selection of that drug would be affected by the presence of a given comorbid symptom occurring with OCBs. Comorbid symptoms queried included depression, anxiety, psychotic behaviors, aggressive or threatening behaviors, impulsivity, insomnia, or hypersexuality. Given comorbid depression, anxiety, impulsivity, or hypersexuality, SSRI drugs were most frequently chosen. Antipsychotic drugs were chosen when comorbid psychotic or aggressive behaviors occurred.  See figure 4 and table 5.

**Figure:**
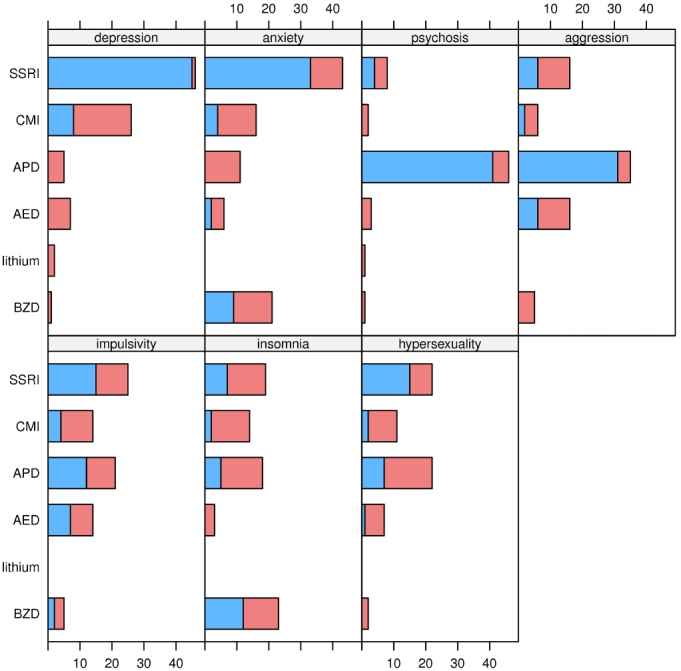
**Figure 4. Choice of drug for treating OCBs that occur with a given comorbid symptom. Blue bars indicate the number of experts who selected the drug as first choice; red bars indicate the number who selected the drug as alternative monotherapy. See box 3 for abbreviations.**

**Table 5. Choice of drug for treating OCBs that occur with a given comorbid symptom. Percentages are relative to the number of experts who provided information for any symptom x drug combination (47). The last column is the sum of the previous two; the percentages do not always match precisely because of roundoff. The table only includes drugs chosen by 10% or more of the experts. See box 3 for abbreviations.**

**Table:** 

				
**depression**	**SSRI**	96%	2%	98%
	**CMI**	17%	38%	55%
	**AED**	0%	15%	15%
	**APD**	0%	11%	11%
**anxiety**	**SSRI**	70%	21%	91%
	**BZD**	19%	26%	45%
	**CMI**	9%	26%	34%
	**AED**	4%	9%	13%
	**APD**	0%	23%	23%
**psychosis**	**APD**	87%	11%	98%
	**SSRI**	9%	9%	17%
**aggression**	**APD**	66%	9%	74%
	**SSRI**	13%	21%	34%
	**AED**	13%	21%	34%
	**CMI**	4%	9%	13%
	**BZD**	0%	11%	11%
**impulsivity**	**SSRI**	32%	21%	53%
	**APD**	26%	19%	45%
	**AED**	15%	15%	30%
	**CMI**	9%	21%	30%
	**BZD**	4%	6%	11%
**insomnia**	**BZD**	26%	23%	49%
	**SSRI**	15%	26%	40%
	**APD**	11%	28%	38%
	**CMI**	4%	26%	30%
**hypersexuality**	**SSRI**	32%	15%	47%
	**APD**	15%	32%	47%
	**CMI**	4%	19%	23%
	**AED**	2%	13%	15%

## **Discussion**

 OCBs are problematic and deserve to be addressed in any comprehensive plan of care for a patient with HD. The high frequency of these symptoms in HD is not surprising, inasmuch as the distinctive neuropathological changes seen in HD (striatal degeneration) are similar to those hypothesized to underlie primary obsessive-compulsive disorder (OCD), namely dysfunction of cortico-striatal connections [17] [18]. Dysfunction in the orbitofrontal cortex, which has extensive connections to the basal ganglia, has been reported in brain imaging studies of individuals with OCD [19] [20], while functional imaging studies have implicated both striatal and cortical abnormalities in patients with primary OCD. Post-pharmacological treatment studies suggest that normalization of caudate and orbitofrontal cortex activation [21] [22] occurs following remission of OCD symptoms.

**Treatment studies**: Formal studies to guide treatment of OCBs in HD are scarce. A Cochrane review of symptomatic treatment for HD concluded that no definitive recommendations could be made for treatment of any behavioral symptoms in HD, due to the lack of controlled studies [23]. Case studies of individual patients report some amelioration of OCBs with various medications including fluoxetine [24], sertraline and olanzapine [25], or olanzapine alone [26]. Despite the probable high frequency of these symptoms as described above, a survey of medication choices for 2128 HD patients showed that less than 2% were being prescribed medications specifically for OCBs [27].   

**Box 3. Key behavioral interventions for managing OCBs.**

**Table:** 

**Expectations**	It is important that family members and other care partners have appropriate expectations regarding a patient's abilities and needs. Some HD patients with high levels of symptomotology have great difficulty controlling OCBs, and should not be expected to control their symptoms. They may not respond quickly and consistently to strategies listed below.
**Prevention**	If there are situations that evoke perseverative behaviors (e.g., discussing driving ability or cigarette smoking), then it is best to avoid these topics.
**Redirection**	Redirection is the most common environmental strategy used. It may take the form of changing the subject, starting a new activity, moving to a different room, placing an interesting object (e.g., a coin) in the patient's hand as a distraction, and the like.
**Setting limits**	It is sometimes useful to set a limit to the perseverative activity and then insist on an end to the activity; however, this is unlikely to work for severely impaired patients or those with extremely poor insight.
**Dramatic termination**	It may be useful to dramatically end a topic or activity. For example, write the topic on a card, and tear up the card, saying, "We are done with that; it's over; no more!" and then move on to a new activity.
**Gradually modifying the activity**	One possibility is for care partners to enter the activity with the patient and gradually add activities to redirect the behavior. For example, a patient who perseverates on rearranging objects in the house could gradually be directed to dusting the room.
**Ignoring**	If perseverative behavior has developed to gain attention from care partners or others, ignoring the behavior will stop reinforcing it positively. However, ignoring perseveration can lead to aggression or outbursts from patients who are frustrated, and may not be useful in many people with HD.
*Source*: LEARNet Tutorial on Perseveration	

**Behavioral interventions**: Most of the experts surveyed agreed that behavioral interventions for patients with mild cognitive impairment, and education for family and care partners are helpful in managing OCBs. However, there is no formal guidance on how to use these interventions in HD. We recommend several strategies, based on treatments used in traumatic brain injury. Box 3 summarizes particularly helpful recommendations.

**Cognitive behavioral therapy**: Cognitive behavioral therapy (CBT), which has established efficacy in primary OCD (APA practice guidelines for OCD, 2007) was endorsed by some of our experts. However, there have been no controlled studies of CBT in HD, and its usefulness may be limited by a patient’s lack of insight or cognitive deficits. Although a large number of our experts had no experience with using CBT for people with HD, those who did thought it could be somewhat useful in HD patients with mild cognitive impairment. 

**SSRIs and CMI**: In general, there was good agreement among the experts surveyed as to first choice of medication for treatment of OCBs Most respondents endorsed SSRIs as the drug of first choice, with clomipramine as a second option. Though fewer had experience with CMI, perceived efficacy of SSRIs and clomipramine was similar. Clomipramine is the tricyclic antidepressant with the highest serotonergic effects, in addition to its effects on norepinephrine. The authors suggest that efforts should be made to increase familiarity with using this medication among HD experts, because clomipramine has good efficacy for treatment of OCBs in the general population (APA OCD practice guidelines, 2007). Clomipramine was listed separately from other TCAs because of its particular efficacy for treatment of OCBs and its FDA approval for treatment of obsessive compulsive disorder. Other TCAs are not nearly as useful for OCBs, as was reflected in responses to survey questions on efficacy.

**Others**: Olanzapine was the top antipsychotic chosen among respondents to this survey, mostly for augmentation use. It is possible that the selection of olanzapine as antipsychotic of choice by HD experts was influenced to some extent by its utility for treating other symptoms of HD, notably irritability and aggression. APA guidelines do not recommend a specific neuroleptic for use in OCD at this time.

**Recommended dosing titration intervals**: Dosing titration interval was an area in which the authors disagree with survey results for some situations. For both SSRI and CMI, the top two pharmacological agents for monotherapy, the majority of respondents reported they would increase from initial dose after 4-6 weeks, followed by 2-4 weeks as the second most endorsed option. The authors feel that dosing changes can be made in 1-2 week intervals. The highest dosing level would be either the top dose recommended by the manufacturer, or the dose best tolerated. More rapid dose escalations are particularly important for treatment of OCBs since these behaviors, like anxiety disorders in general, require longer treatment periods at effective dose for pharmacological treatment to be successful. In the authors' experience, treatment requires going to the highest recommended or tolerated dose.

  Based on the results of this international expert survey, a clinical practice algorithm for the treatment of OCBs in HD was constructed. The authors do not mean to imply that following the steps most often chosen by experts will result in best outcomes,  Treatment response varies greatly in HD, and is particularly hard to predict. The algorithm steps are meant to guide, not decide any individual's treatment.  Only the clinician can address the complexities of any specific patient, where treatment must be tailored to fit individual needs.  

## **Algorithm**

(Click on the figure below for a printable, single page view of the algorithm).

**Figure:**
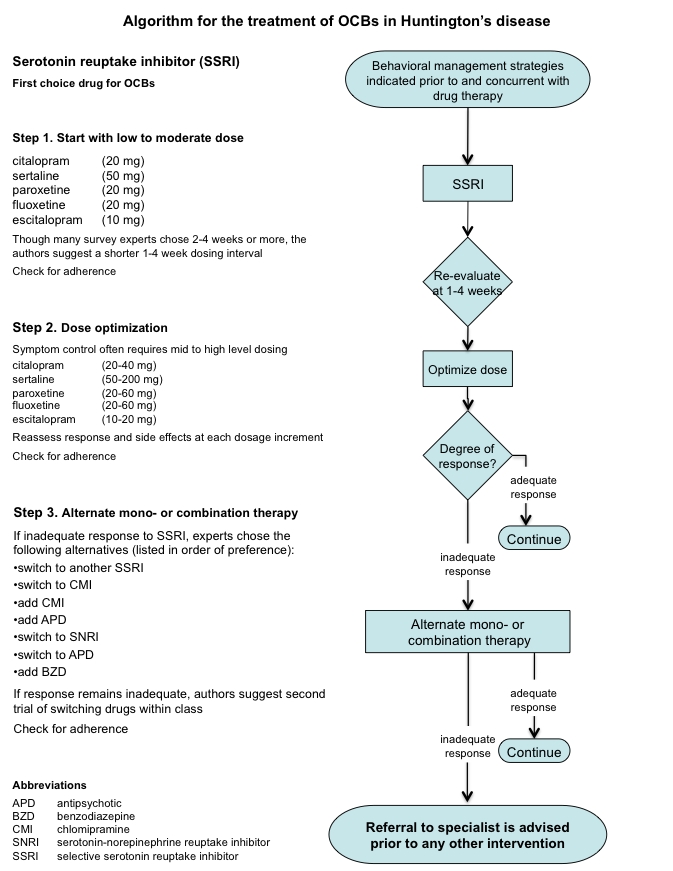

## **Conclusions**

Most experts agreed that behavioral interventions for patients with mild cognitive impairment, and education for family and care partners are helpful in management of OCBs. Further, there was good agreement among the experts that an SSRI is first choice of drug for treatment of OCBs, with CMI as a second option. SSRIs and CMI were perceived as similar in efficacy by the majority of respondents who answered these questions. However, many respondents indicated a lack of experience with CMI or other drug options. Both APDs and AEDs were utilized as augmenting agents when an SSRI or CMI was ineffective alone. There was agreement that BZDs are not substantially effective as monotherapy, but agreement on their use as adjunctive therapy, particularly if anxiety is a comorbid factor.

 The results of the survey point out the need for further study of OCBs in HD. A problem with study of OCBs is that there is not a good clinical or research definition of these behaviors. Development of a standard definition of these symptoms, along with a validated scale for assessment, would greatly advance understanding of these conditions. Review of the literature shows there is a pressing need for treatment studies to determine which psychopharmacological and behavioral treatments are most efficacious for OCBs. Ethically, a placebo-controlled study of medications would be problematic, given that many of the experts surveyed felt several agents had reasonable efficacy for these symptoms. Head to head comparisons of the most frequently used agents would provide practical information without depriving some patients of treatments that are felt to be useful. 

**Limitations**: Survey results are not a substitute for evidence-based study. Instead, these results present treatment options based on a synthesis of opinions from a large group of experts. However, selection of the experts surveyed was not systematic. The core group authors generated a list of expert clinicians based on their personal knowledge of individuals active in the clinical research networks. A systematic survey of all members of the European Huntington's Network and the Huntington Study Group, though less feasible, would have overcome this problem and provided a larger and more diverse sample. As shown in this survey, practice patterns are influenced geographic location. Recall bias may also have occurred, with survey results limited by the accuracy of respondents’ recall, with potential for over- or underestimation of both drug efficacy and side effect frequency. SSRIs were listed first in the questionnaire, reflecting the authors’ own views but possibly biasing answers toward this class of medication. A random order of presentation for medication class, varied among participants, would have been methodologically more sound. Respondents were not asked about use of SNRIs as an independent class of medication, only about their use as second or third line choice or as an augmenting agent. Though we believe the survey questions were comprehensive, they did not cover every possibility and may have omitted other useful queries.

 This project received funding support in part by Lundbeck Inc., an arrangement that could introduce bias. In an effort to limit this bias, HSG and EHDN core committee members and survey respondents had no knowledge of Lundbeck Inc. support during the survey process or data analysis. 

## **Acknowledgements**

 The authors thank those HD experts who shared knowledge and participated in this survey: Karen Anderson, Tomasin Andrews, Kevin Biglan, Jodi Cori-Bloom, Raphael Bonelli, Jean-Marc Burgunder, Jang Ho Cha, Edmond Chiu, Peter Como, Merit Cudkowicz. Matthias Dose, Erik van Duijn, Erik van Duijn, Mary Edmondson, Andy Feigen, Joaquim Ferreira, Mark Groves, Marc Guttman, Don Higgins, Stephen Hersch, Joseph Jankovic, Karl Kieburtz, Barry Kremer, Pierre Krystkowiak, Martin Kucharik, Blair Leavitt, Ann Catherine Bachoud-Levi, Wayne Martin, Elizabeth McCusker, Marsha Nance, Michael Orth, Oksena Osuchowersky, Susan Perlman, Asa Petersen, Josef Priller, Hugh Rickards, Raymund Roos, Adam Rosenblatt, Diana Rosas, Ann Rosser, Jan Roth, Burton Scott, Kathleen Shannon, Shiela Simpson, Ira Shoulson, Nicholas Stoy, Sarah Tabrizi, Francis Walker, Eric Wexler, Vicki Wheelock, Olga Yastrubetskaya, Olga Yastrubetskaya, Daniel Zielonka. 

 Additional gratitude to Dr. Richard Dubinsky and Dr. Eric Wexler who provided expert advice in survey creation, CHDI Foundation for expert advice and technical assistance, and Ann Covalt for editorial assistance. 

## **Funding information**

The Huntington’s Disease Society of America (HDSA), Huntington Society of Canada (HSC), European Huntington’s Disease Network (EHDN), and HD Drug Works (HDDW) provided funding for this project. Support from Lundbeck Inc. was provided by a one time unrestricted grant to HDDW. The combined funds from HDSA, HSC, EHDN, and HDDW provided stipend reimbursement for expert participation. To prevent bias, experts were kept unaware of the Lundbeck Inc. grant.

## **Competing interests**

Dr. Goodman received unrestricted grant and consultant fee support from Lundbeck, Inc. in 2009.  Dr. Edmondson has received consultant fee support from Lundbeck, Inc.

**Author roles**

- Drs. Karen Anderson, HSG core group member, and David Craufurd, EHDN core group member, shared equally in construction and review of survey questionnaire, review of data analysis, writing of first draft of manuscript, and review of manuscript.
- Drs. Mark Groves, HSG Core group member, and Erik van Duijn. EHDN Core group member. Construction and review of survey questionnaire. Review of data analysis. Review of manuscript.
- Dr. Mary Edmondson.  Review of data analysis and manuscript.
- Dr. Dan van Kammen.  Expert adviser. Review of data analysis and manuscript.
- Dr. Nathan Goodman.  Data analysis.
- Dr. LaVonne Goodman. Conception, organization and facilitator for execution of the project. Review of manuscript.
